# Usefulness of preoperative Short Form-36 Mental Component Score as a prognostic factor in patients who underwent decompression surgery for degenerative lumbar spinal stenosis

**DOI:** 10.1097/MD.0000000000030231

**Published:** 2022-09-30

**Authors:** Sangbong Ko, Wonkee Choi

**Affiliations:** a Department of Orthopaedic Surgery, School of Medicine, Daegu Catholic University Hospital, Daegu, Korea

**Keywords:** functional outcome, prognosis, spinal stenosis

## Abstract

Physical factors such as frequency of low back pain, sensory abnormalities in the lower extremities, smoking history before surgery, and preoperative mental health status as predictors of operative outcomes have been growing as areas of interest in the field of degenerative lumbar spinal stenosis (DLSS). This study aimed to investigate the correlation between the preoperative Short Form-36 Mental Component Score (SF-36 MCS) and long-term prognosis after decompression surgery for DLSS. In total, 198 patients were enrolled in this study. The Oswestry Disability Index (ODI) and Rolland Morris Disability Questionnaire (RMDQ) were used to evaluate spinal functional outcomes. The SF-36 questionnaire was used and analyzed by classifying it into physical component score (PCS) and mental component score (MCS). The SF-36 MCS was divided into role limitations caused by emotional problems, social functioning, vitality, and emotional well-being. In the correlation between preoperative MCS and ODI improvement, the *r* value was −0.595 (*P* < .05) at 12 months postoperatively. ODI improvement at 12 months after decompression surgery showed a statistically significant and strong negative correlation with preoperative MCS. In the correlation between preoperative MCS and RMDQ improvement, the *r* value was −0.544 (*P* < .05) at 12 months postoperatively. Therefore, RMDQ improvement 12 months after decompression surgery showed a strong negative correlation with preoperative MCS. Regarding the correlation between preoperative MCS and SF-36 PCS improvement, the *r* values were 0.321 (*P* < .05) at 6 months postoperatively and 0.343 (*P* < .05) at 12 months postoperatively. Therefore, SF-36 PCS improvement at 6 and 12 months after decompression surgery showed a strong positive correlation with preoperative SF-36 MCS scores. Preoperative SF-36 MCS is a factor that can predict the prognosis of patients who underwent decompression surgery for lumbar spinal stenosis for at least 1 year postoperatively.

## 1. Introduction

Degenerative lumbar spinal stenosis (DLSS) is a representative degenerative spine disease that causes neurogenic intermittent claudication (NIC), lower leg radiating pain (LLRP), sensory deficit, motor deficit, and low back pain (LBP) caused by compression of the cauda equina or nerve root.^[1]^ Its prevalence is increasing due to population aging, and it is the most common indication for spinal surgery in patients over the age of 65 years.^[2]^ When the back is extended, the pressure on the cauda equina increases, and the symptoms worsen; when the back is bent, the symptoms are relieved. Acute and severe neurological deficits occur extremely rarely, progress slowly, and cause loss of bodily functions; therefore, conservative treatment is usually employed.^[3]^ However, when conservative treatments fail, decompression of the stenotic segment, which is one of the standardized treatments, can be performed.^[2]^ Preoperative symptoms, such as LLRP and NIC but not LBP, are the best indications for decompression surgery.^[4]^ Decompression surgery is expected to improve these symptoms.^[5,6]^ Several studies also found that one-third of patients were not satisfied with the results owing to residual LLRP, LBP, decreased functional outcome, and poor health-related quality of life (QoL) after decompression surgery.^[7,8]^ Therefore, selecting a patient by understanding their prognostic factors before decompression surgery is essential.

Preoperative mental health status as a predictor of operative outcomes is a growing area of interest. Recently, many studies have reported the use of preoperative mental health status as a predictor of functional outcome and postoperative surgical satisfaction following orthopedic surgery of the upper and lower extremities and the spine.^[9–11]^ Some studies have reported that depression, anxiety, optimism, and overall psychological factors are associated with the functional outcome of spinal surgery and patient satisfaction with surgery.^[12–14]^ Oswestry Disability Index (ODI) and Rolland–Morris Disability Questionnaire (RMDQ) are frequently used for the evaluation of spinal functionality, and the Short Form-36 (SF-36) questionnaire is frequently used for the evaluation of QoL by many spine surgeons. The mental component score (MCS) of this questionnaire decreases as the mental health of the patient declines,^[15]^ and studies have also found that it is an indirect indicator of depressed mental health in patients.^[16]^ To the best of our knowledge, no studies have confirmed whether the preoperative Short Form-36 Mental component score (SF-36 MCS), which evaluates the status of preoperative QoL, can be a prognostic factor following decompression surgery.

Therefore, we investigated the correlation between the MCS of the preoperative SF-36 questionnaire and how it reflects the postoperative physical component score (PCS) and functional outcomes in patients. Moreover, the results of this study may help spine surgeons determine the most important preoperative prognostic factors in patients who would benefit the most from surgery and help achieve satisfactory functional outcomes in preoperative patients.

## 2. Materials and Methods

### 2.1. Study design and populations

This retrospective observational cohort study of prospectively collected data was approved by our institutional review board (IRB number: CR-21-071) and conducted in accordance with the declaration of Helsinki. The data supporting this study are not publicly available because of patient privacy. The requirement for informed consent from the patients was waived because of the retrospective design of this study. A total of 198 patients with DLSS who underwent decompression surgery without fusion between March 2010 and April 2019 were enrolled in this study. All the patients met the inclusion criteria (Table 1).

**Table 1:** Inclusion and exclusion criteria.

Inclusion criteria	
1.	Patients who underwent level 1–2 decompression surgery for DLSS in our hospital
2.	Patients who completed questionnaires for evaluation of functional outcome and quality of life
3.	Patients with at least 1 year of follow-up
Exclusion criteria	
1.	Patients who required additional instrumentation, fusion and revision surgery other than decompression surgery
2.	Patients with pre-existing psychiatricmedical history

### 2.2. Methods

In our hospital, patients who underwent decompression surgery for level 1–2 DLSS were evaluated preoperatively at baseline and were followed up for at least 1 year postoperatively. To evaluate spinal functional outcomes, the Oswestry Disability Index (ODI) and Rolland Morris Disability Questionnaire (RMDQ), which indicate better functional outcomes with increased numerical values, were used. The difference between the postoperative and preoperative scores was determined as the degree of improvement and was analyzed. The SF-36 questionnaire, a tool for evaluating the self-reported QoL of patients, was used and analyzed by classifying it into PCS and MCS. The SF-36 PCS, in which higher scores indicate better health, was analyzed by determining the difference between the postoperative and preoperative states as the degree of improvement. Moreover, the SF-36 MCS was divided into role limitations caused by emotional problems (RE), social functioning (SF), vitality (VT), and emotional well-being (MH), and the association between these parameters and postoperative improvement was evaluated.

### 2.3. Statistical analysis

Continuous data are expressed as mean and standard deviation. Pearson’s test was used to analyze the correlation between the preoperative SF-36 MCS and 3 subgroup (SF, VT, and MH) scores and the degree of improvement in functional outcome and QoL at 3, 6, and 12 months postoperatively. Pearson’s *r* value shows an extremely strong negative relationship between −1.0 and −0.7, a strong negative relationship between −0.7 and −0.3, a weak negative relationship between −0.3 and −0.1, and no correlation between −0.1 and 0.1. Moreover, 0.1–0.3 was considered a weak positive correlation, 0.3–0.7 was considered a strong positive correlation, and 0.7–1.0 was considered an extremely strong positive correlation. Preoperative RE values of SF-36 MCS were evaluated as 0, 33.3, 66.6, and 100, so they were not evaluated as continuous variables and were divided into 4 groups. The postoperative functional outcome was analyzed using the analysis of variance (1-way ANOVA), followed by the Bonferroni test for post hoc analysis. Statistical significance was set at *P* value <.05.

## 3. Results

### 3.1. Epidemiological characteristics of all participants

The mean age of the 198 patients (58 men, 140 women) was 69.63 ± 8.11 years. In the preoperative evaluation of spinal functional outcome, the mean ODI was 25.38 ± 8.34, the mean RMDQ was 13.42 ± 5.60, the SF-36 PCS, which is the patient’s QoL evaluation, was 22.04 ± 12.56, and the SF-36 MCS was 35.90 ± 17.65. The SF-36 MCS scores were 13.81 ± 30.60 for RE, 42.44 ± 28.40 SF, 39.92 ± 19.27 VT and 49.09 ± 18.66 for MH (Table 2).

**Table 2:** Epidemiological characteristics of all participants.

Baseline data			
Sex ratio			M/F(58/140)
Age			69.63 ± 8.11
Functional Outcome	ODI		25.38 ± 8.34
	RMDQ		13.42 ± 5.60
SF-36	PCS		22.04 ± 12.56
	MCS	RE	13.81 ± 30.60
		SF	42.44 ± 28.40
		VT	39.92 ± 19.27
		MH	49.09 ± 18.66

F = female, M = male, MCS = Mental Component Score, MH = emotional well-being, ODI = Oswestry Disability Index, PCS = Physical Component Score, RE = role limitations due to emotional problems, RMDQ = Rolland Morris Disability Questionnaire, SF = social functioning, SF-36 = Short Form-36, VT = vitality.

### 3.2. Correlation between preoperative SF-36 MCS and improvement in postoperative ODI

The mean degrees of ODI improvement postoperatively were 3.90 ± 9.34 at 3 months, 8.99 ± 9.93 at 6 months, and 11.39 ± 12.68 at 12 months. In correlation with preoperative MCS, the r values postoperatively were −0.136 (*P* = .057) at 3 months, −0.181 (*P* = .011) at 6 months and −0.595 (*P* < .05) at 12 months. In the MCS subgroup, the *r* values for ODI improvement at 3 months were −0.193 for SF (*P* = .006), −0.020 for VT (*P* = .782), and −0.099 for MH (*P* = .167), and those for ODI improvement at 6 months were −0.268 for SF (*P* < .05), −0.019 for VT (*P* = .792), and 0.008 for MH (*P* = .914). Moreover, the r values for ODI improvement at 12 months were −0.604 for SF (*P* < .05), −0.510 for VT (*P* < .05) and −0.432 for MH (*P* < .05). Therefore, ODI improvement at 12 months after decompression surgery showed a statistically significant and strong negative correlation with preoperative MCS and SF, VT, and MH subclasses (Table 3).

**Table 3:** Correlation between preoperative SF-36 MCS and improvement in postoperative ODI.

			ODI 3	ODI 6	ODI 12
MCS		Pearson’s *r*	−.136	−.181*	−.595**
		*P* value	.057	.011	.000
	SF	Pearson’s *r*	*−*.193**	−.268**	−.604**
		*P* value	.006	.000	.000
	VT	Pearson’s r	−.020	−.019	−.510**
		*P* value	.782	.792	.000
	MH	Pearson’s *r*	−.099	.008	−.432**
		*P* value	.167	.914	.000

Bold values indicate statistical significance, italic values indicate strong negative correlation.

MCS = Mental Component Score; MH = emotional well-being; ODI 12 = 12-month ODI improvement; ODI 3 = 3-month ODI improvement; ODI 6 = 6-month ODI improvement; ODI = Oswestry Disability Index; SF = social functioning; SF-36 = Short Form-36; VT = vitality.

### 3.3. Correlation between preoperative SF-36 MCS and improvement in postoperative RMDQ

The mean postoperative improvement in the RMDQ was 2.212 ± 6.432 at 3 months, 5.182 ± 5.692 at 6 months, and 6.525 ± 8.822 at 12 months. In correlation with preoperative MCS, the *r* values postoperatively were −0.047 (*P* = .511) at 3 months, −0.087 (*P* = .23) at 6 months and −0.544 (*P* < .05) at 12 months. In correlation with the MCS subgroup, the r values for RMDQ improvement at 3 months were −0.087 for SF (*P* = .225), −0.121 for VT (*P* = .090) and −0.130 for MH (*P* = .067). The *r* values for RMDQ improvement at 6 months were −0.264 for SF (*P* < .05), −0.042 for VT (*P* = .557), and −0.070 for MH (*P* = .329), whereas those for RMDQ improvement at 12 months were −0.475 for SF (*P* < .05), −0.569 for VT (*P* < .05), and −0.531 for MH (*P* < .05). Therefore, RMDQ improvement at 12 months after decompression surgery showed a strong negative correlation with preoperative MCS and SF, VT, and MH subclasses (Table 4).

**Table 4:** Correlation between preoperative SF-36 MCS and improvement in postoperative RMDQ.

			RMDQ 3	RMDQ 6	RMDQ 12
MCS		Pearson’s *r*	−.047	−.087	−.544**
		*P* value	.511	.223	.000
	SF	Pearson’s *r*	−.087	−.264**	−.475**
		*P* value	.225	.000	.000
	VT	Pearson’s *r*	−.121	−.042	−.569**
		*P* value	.090	.557	.000
	MH	Pearson’s *r*	−.130	−.070	−.531**
		*P* value	.067	.329	.000

Bold values indicate statistical significance, italic values indicate strong negative correlation.

MCS = Mental Component Score, MH = emotional well-being, RMDQ = Rolland Morris Disability Questionnaire, RMDQ 12 = 12-month RMDQ improvement, RMDQ 3 = 3-month RMDQ improvement, RMDQ 6 = 6-month RMDQ improvement, SF = social functioning, SF-36 = Short Form-36, VT = vitality.

### 3.4. Correlation between preoperative SF-36 MCS and improvement in postoperative SF-36 PCS

The mean postoperative improvement in the SF-36 PCS was 11.660 ± 23.889 at 3 months, 22.391 ± 27.431 at 6 months, and 27.618 ± 27.417 at 12 months. In correlation with the preoperative MCS, the r values postoperatively were 0.191(*P* = .007) at 3 months, 0.321 (*P* < .05) at 6 months, and 0.343 (*P* < .05) at 12 months. In the MCS subgroup analysis, the *r* values for SF-36 PCS improvement 6 months after decompression surgery were 0.167 for SF (*P* = .019), 0.269 for VT (*P* < .05), and 0.254 for MH (*P* < .05). Moreover, the *r* values for SF-36 PCS improvement at 6 months were 0.217 for SF (*P* = .002), 0.309 for VT (*P* < .05), and 0.336 for MH (*P* < .05), and those for SF-36 PCS improvement at 12 months were 0.381 for SF (*P* < .05), 0.308 for VT (*P* < .05), and 0.096 for MH (*P* = .178). Therefore, SF-36 PCS improvement at 6 and 12 months after decompression surgery showed a strong positive correlation with preoperative SF-36 MCS scores. The SF-36 PCS improvement at 6 months postoperatively showed a strong positive correlation with VT and MH, and the SF-36 PCS improvement at 12 months postoperatively showed a strong positive correlation with SF and VT (Table 5).

**Table 5:** Correlation between preoperative SF-36 MCS and improvement in postoperative SF-36 PCS.

			SF-36 PCS 3	SF-36 PCS 6	SF-36 PCS 12
MCS		Pearson’s *r*	.191**	.321**	−.343**
		*P* value	.007	.000	.000
	SF	Pearson’s *r*	.167*	.217**	−.381**
		P value	.019	.002	.000
	VT	Pearson’s *r*	.269**	.309**	−.308**
		*P* value	.000	.000	.000
	MH	Pearson’s *r*	.254**	.336**	−.096
		*P* value	.000	.000	.178

Bold values indicate statistical significance, italic values indicate strong negative correlation.

MCS = Mental Component Score, MH = emotional well-being, PCS = Physical Component Score, SF = social functioning, SF-36 PCS 12 = 12-month SF-36 PCS improvement, SF-36 PCS 3 = 3-month SF-36 PCS improvement, SF-36 PCS 6 = 6-month SF-36 PCS improvement, SF-36 = Short Form-36, VT, vitality.

### 3.5. Correlation between preoperative RE score of SF-36 MCS and functional outcome

After designating RE values of 0, 33.3333, 66.6666, and 100 to 4 groups of 1, 2, 3, and 4, the degree of functional improvement after surgery was compared using ANOVA. The findings suggested that there was a statistically significant difference in the mean values between the groups in the 3-month postoperative SF-36 PCS, 6-month postoperative ODI, RMDQ, and 12-month postoperative ODI, RMDQ, and SF-36 PCS. Based on the post hoc analysis, in all ODI, RMDQ, and SF-36 PCS at 12 months postoperatively, the degree of improvement in postoperative SF-36 PCS was significantly higher in group 1, with the lowest preoperative RE, than in group 4, with the highest preoperative RE.

## 4. Discussion

Predicting a patient’s functional outcome, prognosis, and postoperative satisfaction is of considerable importance to surgeons. It is particularly important in patients with DLSS because it occurs mainly during the process of degeneration in the elderly population and is often accompanied by other chronic diseases. When LBP is the main symptom, other symptoms such as long-term LLRP or LBP and presence of severe pain, obesity, spinal history, and several medical comorbidities are poor prognostic factors^[8,17,18]^. Factors such as LBP frequency, sensory abnormalities in the lower extremities, preoperative function, QoL, smoking history, and cardiovascular disease are also used for prognosis.^[7,17,18]^

Furthermore, it has recently been argued that the psychological and mental state of the patient is also associated with the prognosis of the patient after decompression surgery. Some studies have suggested that depression results in poor outcomes after spinal surgery, including lumbar decompression surgery for DLSS,^[12,19,20]^ but some studies have reported no such association.^[21,22]^ Miller et al^[19]^ analyzed how much the degree of depression (using the Patient Health Questionnaire 9) was associated with pain and QoL (using the Pain Disability Questionnaire and EuroQoL-five-dimensional) in 919 patients, and reported that worsening depression predicts worse functional outcomes regardless of the diagnosis or type of spinal surgery. Screening for depression before surgery is essential, and preoperative treatment for depression reportedly showed better functional outcomes.^[12]^ Merrill et al^[12]^ also argued that the diagnosis of depression predicts worse outcomes after surgery; therefore, identifying and treating depression before lumbar decompression surgery is crucial. In addition to depression, Dobran et al^[13]^ reported depression, anxiety, and global psychological distress using the Symptom Checklist-90-Revised in 25 patients who underwent decompression or decompression with fusion for lumbar spinal stenosis. Moreover, by analyzing the association between postoperative ODI and pain in patients, preoperative baseline global psychological distress, depression, and anxiety were associated with poorer postoperative clinical outcomes. Kitano et al^[14]^ argued that, in addition to depression, patients’ anxiety levels and catastrophic thinking, which negatively captured pain experience, could affect the postoperative functional outcome. They conducted a Self-Rating Questionnaire for Depression, Hospital Anxiety and Depression Scale, Pain Catastrophic Scale, Pain Anxiety Symptom Scale-20, and a brief class for psychiatric problems in orthopedic patients.

The SF-36 is commonly used by physicians to evaluate a patient’s QoL because of its history, conciseness, reliability, and effectiveness. Its score is largely divided into PCS, which evaluates physical function, and MCS, which evaluates mental function, according to the original guidelines of Ware and Kosinski,^[23]^ and the summary score is always compared with 8 subclass scale scores before concluding. PCS and MCS are relevant to orthopedics because many orthopedic patients have extremely low physical health scale scores on the SF-36 because of their musculoskeletal conditions, resulting in a higher MCS than that implied by the SF-36 scale scores. General health scores, such as those in the SF-36, are designed to provide a more global overview of health and function but have limited sensitivity to small changes in disease progression or specific anatomic regions.^[24]^ Therefore, the authors assumed that the MCS item of the SF-36, which is generally used for evaluating the patient’s QoL, is related to the patient’s psychological status and attempted to investigate the relevance of this to the postoperative functional outcome. In this study, preoperative MCS showed a strong correlation with ODI and RMDQ improvement at 12 months postoperatively, and with PCS improvement at 6 and 12 months postoperatively. Patients who reported the most severe pain and disability preoperatively achieved the highest gains in function and pain relief following surgical treatment at an average of 5-year postoperative follow-up. This is consistent with our previous analysis of the factors that affect the magnitude of change at the 1-year postoperative time point. These findings have been reported for hip, knee, shoulder, and ankle arthroplasty.^[25]^ Lai et al^[9]^ reported that functional outcomes 1 year after hallux valgus surgery were associated with preoperative MCS. Decompression surgery for DLSS performed by the authors was assumed to follow a similar clinical course.

This study has some limitations. First, patients with a higher preoperative MCS status may still have a better postoperative functional outcome owing to a higher preoperative baseline score. Therefore, we did not analyze each value to overcome this limitation but compared the degree of improvement in patients, that is, the degree of improvement from the postoperative to the preoperative state. However, patients with higher MCS scores are more likely to have a higher baseline preoperative functional score and are therefore less likely to achieve dramatic improvements postoperatively, as measured by significant improvements in their functional scores. Second, radiological analysis was not performed. The degree of the initial stenosis, lateral recess stenosis, or central stenosis was not evaluated. Moreover, we did not evaluate the degree of decompression, as we did not attempt to perform additional techniques such as magnetic resonance imaging or computed tomography after surgery. Finally, the advantages of using the PCS and MCS measures rather than the 8-scale scores include smaller confidence intervals and smaller floor and ceiling effects, and a decrease in the number of analyses required from 8 to 2 also avoids some reduction in statistical power resulting from multiple testing.

## 5. Conclusion

The MCS items of the SF-36 questionnaire for evaluating preoperative QoL were correlated with improvement in the spine-related functional outcome at 12 months postoperatively and in the PCS of SF-36 at 6 and 12 months postoperatively in patients with spinal stenosis requiring decompression surgery. Therefore, preoperative SF-36 MCS is one of the factors that can predict the prognosis of patients who have undergone decompression surgery for lumbar spinal stenosis for at least 1 year postoperatively.

## Author contributions

Conceptualization: Sangbong Ko.

Data curation: Sangbong Ko.

Formal analysis: Wonkee Choi, Sangbong Ko.

Funding acquisition: None

Investigation: Sangbong Ko.

Methodology: Sangbong Ko.

Project administration: Sangbong Ko.

Resources: Sangbong Ko.

Software: Sangbong Ko, Wonkee Choi.

Supervision: Sangbong Ko

Validation: Sangbong Ko, Wonkee Choi.

Visualization: Wonkee Choi.

Writing – Original draft: Sangbong Ko, Wonkee Choi.

Writing-review & editing: Sangbong Ko
